# Vascular tortuosity analysis in eyes with epiretinal membrane imaged by optical coherence tomography angiography

**DOI:** 10.1186/s12886-022-02420-z

**Published:** 2022-05-02

**Authors:** Kosuke Miyazawa, Susumu Sakimoto, Masanori Kanai, Akihiko Shiraki, Shizuka Takahashi, Nobuhiko Shiraki, Kazuichi Maruyama, Hirokazu Sakaguchi, Kohji Nishida

**Affiliations:** 1 Department of Ophthalmology, Osaka University Graduate School of Medicine, Rm. E7, 2-2 Yamadaoka, Suita, Osaka, 565-0871 Japan; 2 Integrated Frontier Research for Medical Science Division, Institute for Open and Transdisciplinary Research Initiatives, Osaka University, Suita, Osaka Japan; 3 Department of Ophthalmology, Gifu University Graduate School of Medicine, Yanagido, Gifu, Japan

**Keywords:** Epiretinal membrane, Optical coherence tomography angiography, Vitrectomy, Imaging analysis, Vascular tortuosity

## Abstract

**Background:**

This study aimed to evaluate macular vessel tortuosity using optical coherence tomography angiography (OCTA) and its association with visual outcomes in eyes undergoing surgery for epiretinal membrane (ERM).

**Methods:**

The study included 22 consecutive patients who underwent vitrectomy for ERM between May 2019 and July 2020 and OCTA at Osaka University Hospital. All patients underwent ophthalmologic examinations, including swept-source OCTA. Standard vitrectomy was performed, and the patients were followed up for 6 months postoperatively. Distortion of retinal vessels was calculated using two parameters: the actual vessel length in the vessel section (VL) and the direct vessel branching point distance (BD) in the three quadrants (nasal, temporal, and superior-inferior) of the macula. We analyzed the correlation between these parameters and visual outcomes.

**Results:**

Significantly longer VL was found at 1, 3, and 6 months postoperatively (*p* = 0.006, 0.008, and 0.022, respectively) in the temporal quadrant compared to baseline temporal VL. Significantly shorter VL was found in nasal quadrants at 1 and 3 months (*p* = 0.046 and *p* = 0.018) in the comparison of nasal baseline VL. VL/BDs were correlated with the same postoperative best-corrected visual acuity (BCVA) at 1, 3, and 6 months (*p* = 0.035, 0.035, and 0.042, respectively) in the superior-inferior quadrant. A significant association of changes in VL and BCVA was found at 3 and 6 months postoperatively in the nasal quadrant (*p* = 0.018 and 0.0455, respectively).

**Conclusions:**

Changes in vascular distortion after ERM surgery can be measured using OCTA. The change in vessels around the macula became more linear; this was associated with visual outcomes after surgery.

## Background

Epiretinal membranes (ERMs) are fibrocellular membranes consisting of glial cells, fibroblasts, and extracellular matrix that spread on the inner surface of the retina [1–4]. The traction caused by ERMs alters the macular microstructure and subsequently thickens the macula, leading to the development of ectopic inner foveal layers and disruption of the outer and inner retinal layers [2, 5–7]. The pathogenesis originating from retinal traction involves decreased vision and metamorphopsia [8, 9]. Vitrectomy and membrane peeling, which are the standard procedures for ERM treatment, can normalize the wrinkled retinal surface and thickened macula [2, 10, 11]. Since there is no defined modality to measure the retinal traction caused by ERM, its effect on vision remains to be elucidated. Kofod et al. demonstrated that retinal vessel movement in eyes with ERM correlated with worsening of best-corrected visual acuity (BCVA) and increased central macular thickness (CMT) [12]. For the quantitative evaluation of retinal traction, previous studies have measured the moving distance of retinal vessels caused by ERM contraction [13–15]. Previous studies have also evaluated the parameters by analyzing fundus photographs after ERM surgery to quantify the distance of retinal vessel movement. However, fundus photography is not a suitable modality to visualize retinal vessels.

Optical coherence tomography angiography (OCTA) creates an image of retinal blood flow using the movement of red blood cells. This technology, combined with a B-scan, allows segmented evaluation of macular capillary networks. With these advantages, OCTA should be more efficacious than color fundus photography or fluorescein angiography to investigate the position of retinal vasculature repeatedly in short intervals. Thus, OCTA is a suitable method for evaluating retinal vasculature distortion in eyes with ERM. Moreover, we sought to analyze OCTA images of eyes with ERMs to measure the retinal traction during the postoperative course.

In this study, we quantified the strain on vessels around the macula by measuring the amount of retinal vessel distortion with OCTA for the first time. We hypothesized that after surgery for eyes with ERM, the strain on vessels should be decreased by the release of tractional forces. We utilized OCTA to evaluate and establish methods for assessing vessel compression after ERM surgery. Moreover, we evaluated the correlation between vascular parameters and visual outcomes in ERM surgery.

## Methods

### Subjects

Inclusion criteria were as follows: 1) All procedures were performed at the Department of Ophthalmology in Osaka University Hospital, between May 2019 and July 2020. 2) All patients underwent comprehensive ophthalmological examinations before surgery and at 1, 3, and 6 months after surgery. Examinations included the measurement of BCVA, intraocular pressure, refraction, fundus photographs, spectral-domain OCT (SD-OCT; Cirrus®: Carl Zeiss Meditec Inc., Jena, Germany) and swept-source OCTA (Plex® Elite 9000; Carl Zeiss Meditec Inc., Jena, Germany). Refraction was performed using a Snellen chart, and measurements were recorded by trained optometrists. For statistical analysis, BCVA was converted to logarithm of the minimal angle of resolution. 3) Presence of a unilateral ERM diagnosed by SD-OCT, based on the report by Govetto et al. [5]. Exclusion criteria were association of cataract severe than Emery grade 2, secondary ERM due to any cause, bilateral ERM, macular pseudohole or lamellar hole, association of glaucoma and high myopia (spherical equivalent ≥6.0 diopters or axial length of 26 mm), or other ocular pathologies that could affect visual acuity. This study adhered to the tenets of the Declaration of Helsinki and was approved by the Institutional Review Board of Osaka University Hospital (Japan). All participants provided informed consent.

### OCT and OCTA

The central subfield thickness (CST) was measured using an SD-OCT software (Cirrus® 6000; Carl Zeiss Meditec Inc., Jena, Germany) as an average retinal thickness within a 1-mm circle centered on the fovea. When the CST could not be measured correctly using the software, we measured it manually. OCTA images were obtained using swept-source OCTA. En face images of the retinal vessels were made from the total retina based on automated layer segmentation performed by the software installed in the OCTA device. The foveal avascular zone (FAZ) area was analyzed using ImageJ software [16] to calculate the size of FAZ on en face OCTA images, which captured both the superficial and deep capillary plexus. To avoid segmentation error, manual segmentation was performed to assess the total retina, if needed. Poor-quality images, such as those with poor contrast due to media opacity or poorly fixated images, were excluded.

### OCTA evaluation

**Fig. 1:**
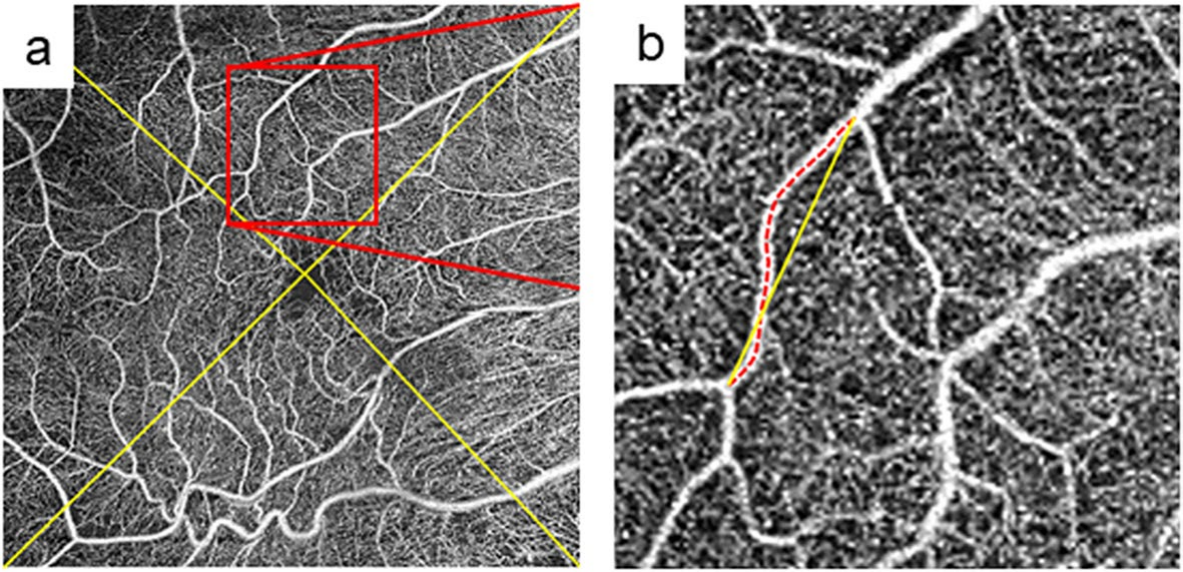
Representative en face image of optical coherence tomography angiography (OCTA) shows microvascular structure around the macula in a right eye with epiretinal membrane. **a** A 6 × 6 mm en face image of OCTA is divided into four quadrants. **b** A high magnification image of the red square in (a). A retinal vessel was randomly picked in each quadrant to macula descending to macula. We also picked two adjacent branch points. Red dotted line represents the actual vessel length in the vessel section and yellow line represents the direct vessel branching point distance between two adjacent branch points

We hypothesized that a decrease in the strain on retinal vessels after ERM removal resulted in an increase in the length of the retinal vessel in the specific segment. Thus, distortion of retinal vessels was calculated using two parameters: the actual vessel length in the vessel section (VL) and the direct vessel branching point distance (BD; Fig. 1). We manually picked a retinal vessel (not capillary) in each of the four quadrants of the vessels descending to the macula. We also selected two adjacent branch points to measure the VL and BD (Fig. 1). VL was defined as the vessel segment length between the two branch points included in each area, and BD was defined as the direct distance between these branch points. VL and BD were calculated semi-automatically from the 6 × 6 mm en face OCTA image of the total retina mode using the ImageJ software. We divided the VL by BD to calculate the distortion of vessels in the four quadrants (VL/BD, Fig. 1). The image observers were blinded to the patient’s symptoms, BCVA, and macular thickness. Preoperative and postoperative (1, 3, and 6 months after surgery) VL and BD were analyzed.

### Surgical techniques

A standard 25-gauge, three-port pars plana vitrectomy was performed by vitreoretinal experts (SS, KN, KM, and HS) using the Constellation vision system (Alcon Laboratories, Inc., Fort Worth, TX, USA). After core vitrectomy, the ERM and internal limiting membrane were peeled circumferentially from the macula with vitreoretinal forceps in all cases. Finally, we performed a careful inspection of the periphery over 360°. Simultaneous cataract surgery was performed in all phakic eyes.

#### Statistics

The data were analyzed using GraphPad Prism (GraphPad Software Inc., La Jolla, CA, USA). One-way analysis of variance, Mann–Whitney U test, paired t-test, and Spearman’s rank correlation coefficient were performed as appropriate. Statistical significance was set at *P* < 0.05.

## Results

A total of 22 patients (7 men and 15 women) with unilateral idiopathic ERM were included in this study, and their mean age was 72.0 ± 8.9 years. Three eyes (13.6%) were pseudophakic at baseline, and the remaining 19 eyes (86.3%) underwent concomitant cataract surgery. The number of eyes in the four ERM stages was as follows: 1 eye (4.5%) in stage 1, 3 eyes (13.6%) in stage 2, 13 (59.1%) in stage 3, and 5 (222.7%) in stage 4. The mean preoperative BCVA was 0.21 ± 0.25 (− 0.18–0.82) and postoperative BCVA at 1, 3, and 6 months were significantly improved compared to the preoperative BCVA as 0.13 ± 0.21 (− 0.18–0.70, *p* = 0.026), 0.08 ± 0.19 (− 0.18–0.52, *p* = 0.001), and 0.02 ± 0.19 (− 0.18–0.40, *p* < 0.001), respectively. Preoperative and postoperative CMT or FAZ area are shown in Table 1.

**Table 1:** Patient characteristics

Parameter	Value
Mean age ± SD, yrs	72.05 ± 8.86 (57–88)
Gender, n (male/female)	7/15
Axial length (mm)	23.55 ± 0.87 (22.51 to 25.71)
Preoperative phakic / IOL	19/3
ERM stage 1:2:3:4	1:3:13:5
Internal Limiting Membrane Dye	
Triamcinolone	17
Indocyanine green	5
Preoperative logMAR BCVA	0.21 ± 0.25(−0.18 to 0.82)
Postoperative logMAR BCVA	
1 month	0.13 ± 0.21(−0.18 to 0.70)
3 months	0.08 ± 0.19(−0.18 to 0.52)
6 months	0.02 ± 0.19(− 0.18 to 0.40)
Preoperative central macular thickness (μm)	431.0 ± 98.4 (260 to 628)
Postoperative central macular thickness (μm)	
1 month	412.0 ± 52.6 (333 to 498)
3 months	390.6 ± 49.3 (308 to 496)
6 months	383.1 ± 45.1 (316 to 496)
Preoperative foveal avascular zone area (mm^2^)	0.081 ± 0.068 (0.020 to 0.300)
Postoperative foveal avascular zone area (mm2)	
1 month	0.074 ± 0.035 (0.025 to 0.136)
3 months	0.079 ± 0.037 (0.028 to 0.152)
6 months	0.079 ± 0.037 (0.029 to 0.161)

*SD* Standard deviation, *IOL* Intraocular lens, *ERM* Epiretinal membrane, *logMAR* Logarithm of the Minimum Angle of Resolution, *BCVA* Best-corrected visual acuity

**Fig. 2:**
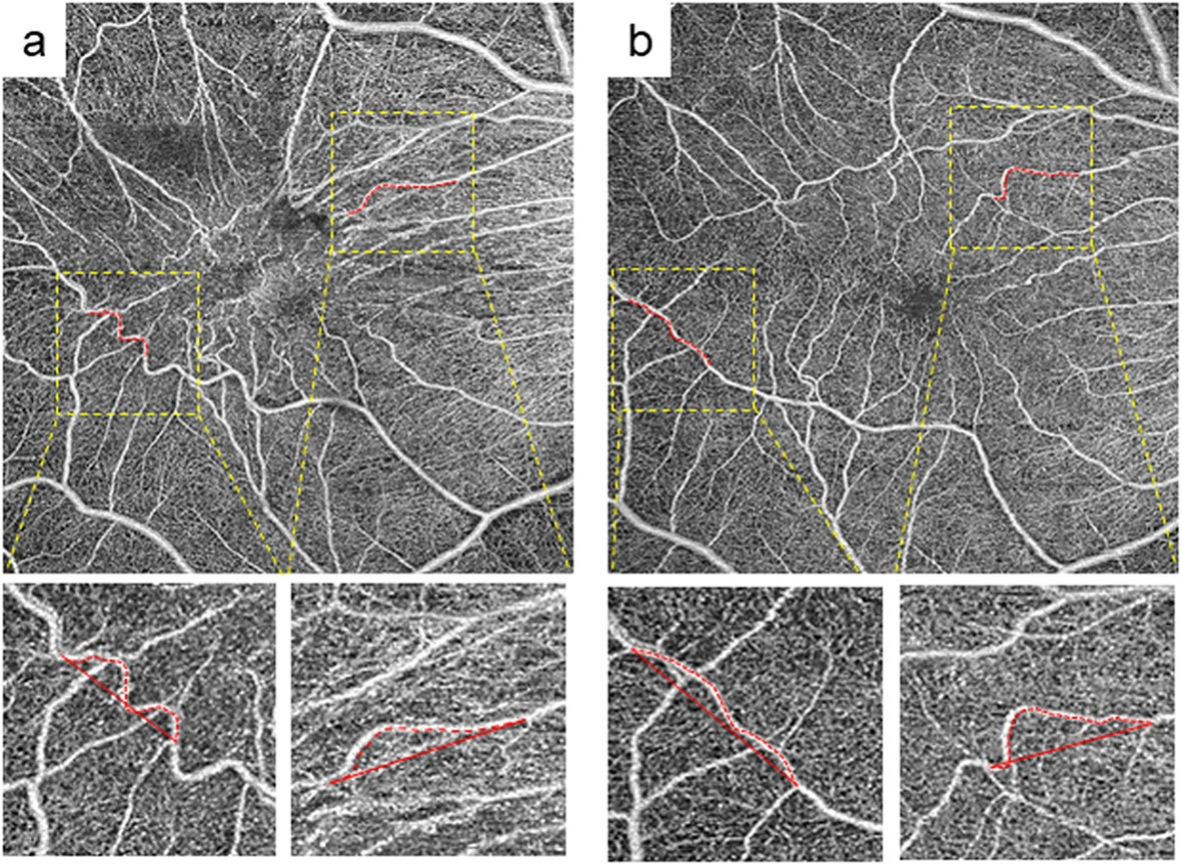
Representative en face images of preoperative and postoperative optical coherence tomography angiography (OCTA) in eyes with ERM. **a** Preoperative and **b** postoperative 1 month 6 × 6 mm OCTA images. Red dotted lines show temporal and nasal vessels analyzed in this study. High magnification images are shown below, which correspond to the dotted yellow square. The length of red dotted lines defined as “the actual vessel length (VL)” and the length of red lines defined as “the direct vessel branching point distance (BD),” respectively. Note that VL becomes shorter in temporal vessels and longer in nasal vessels postoperatively. In contrast, BD becomes longer in temporal and shorter in nasal postoperatively

To assess the vascular strain caused by ERM traction, we quantified VL, BD, and VL/BD. Baseline and postoperative VL, BD, and VL/BD in the superior-inferior, nasal, and temporal quadrants are shown in Table 2. Significantly longer VL was found in the temporal quadrant at 1, 3, and 6 months (*p* = 0.006, 0.008, and 0.022, respectively) compared to baseline VL. Significantly shorter VL was found in the nasal quadrants at 1 and 3 months (*p* = 0.046 and 0.018, respectively) (Table 2) (Fig. 2A and B) compared to baseline VL. Significantly longer BD was noted in three quadrants at 1, 3, and 6 months postoperatively [superior-inferior (*p* < 0.001, < 0.001, and < 0.001, respectively) and temporal quadrants (*p* < 0.001, 0.002, and 0.001, respectively) but in the nasal quadrants, significantly shorter BD was noted at 1, 3, and 6 months postoperatively (*p* = 0.005, 0.011, and 0.027, respectively)] compared to baseline BD. In contrast, a significant decrease in VL/BD compared to baseline was observed at 1, 3, and 6 months in the superior-inferior quadrant (*p* = 0.002, *p* < 0.001, and *p* < 0.001, respectively) and in the temporal quadrant (*p* = 0.037, 0.019, and 0.012, respectively); however, no significant change in VL/BD was observed in the nasal quadrant (*p* = 0.092, 0.181, and 0.135, respectively).

**Table 2:** Vascular changes measured by OCTA

The actual vessel length in area (VL) (um)	Value	*p* value
Temporal		
preoperative	1375.9 ± 577.9	
1 M	1421.2 ± 547.7	0.006
3 M	1429.3 ± 550.6	0.008
6 M	1419.8 ± 564.9	0.022
Nasal		
preoperative	1147.8 ± 293.6	
1 M	1111.5 ± 267.9	0.046
3 M	1105.7 ± 274.1	0.018
6 M	1115.5 ± 270.5	0.083
Superior + Inferior		
preoperative	1322.6 ± 509.6	
1 M	1344.6 ± 501.0	0.052
3 M	1342.8 ± 499.6	0.061
6 M	1344.5 ± 493.3	0.053

*OCTA* Optical coherence tomography angiography, *1 M* 1 month, *3 M* 3 months, *6 M* 6 months

**Fig. 3:**
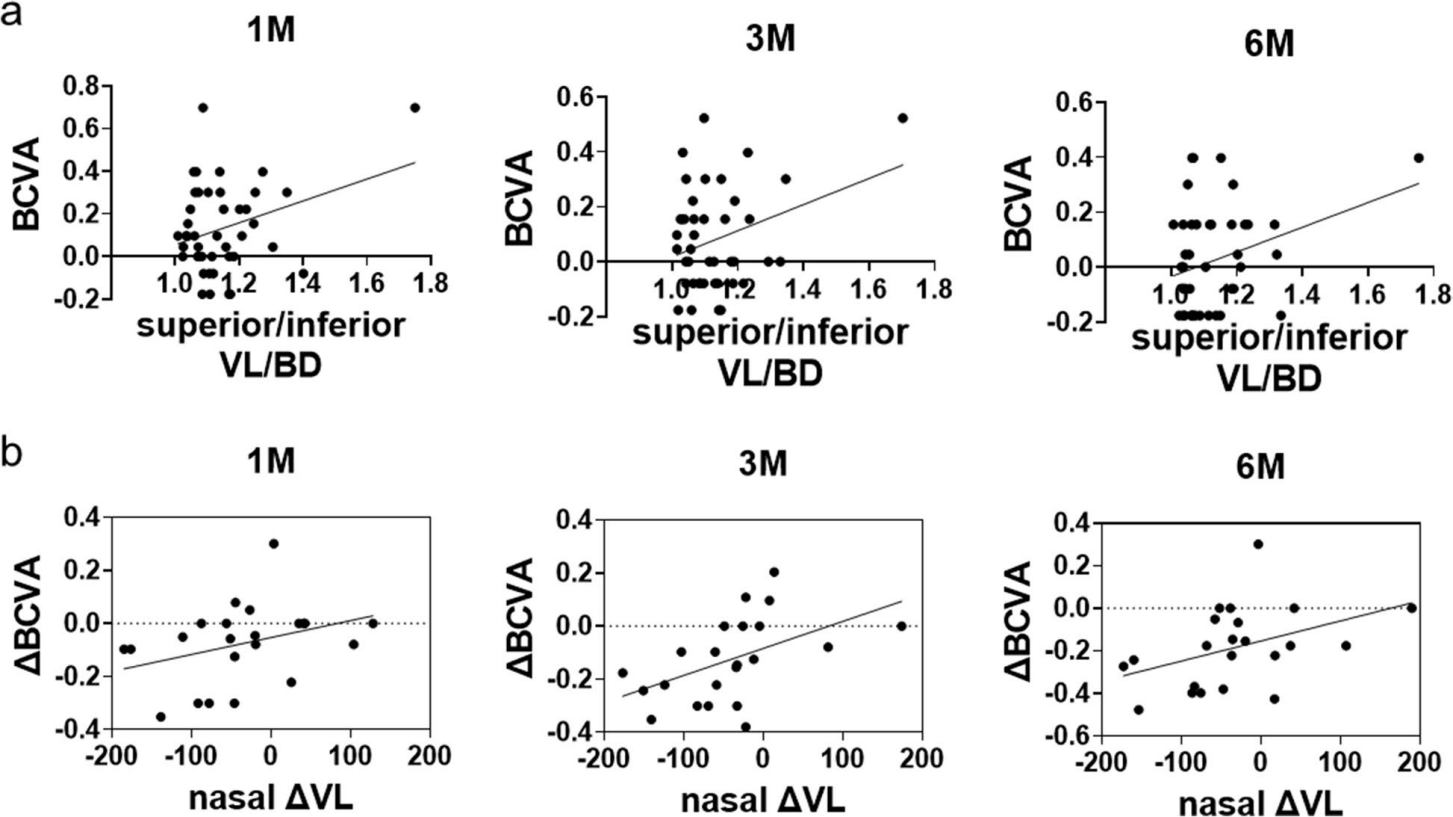
Scatter plots of data summary described in Tables 3 and 4. **a** Scatter plots depicting the association between superior/inferior VL/BD and BCVA postoperatively at 1 month (1 M), 3 months (3 M), and 6 months (6 M), which was shown in Table 3. Lines show regression lines. **b** Scatter plots depicting the association between the change of VL in the nasal quadrant and the change of BCVA postoperatively at 1 month (1 M), 3 months (3 M), and 6 months (6 M), which was shown in Table 4. Lines show regression lines. VL: the actual vessel length, BD: the direct vessel branching point distance, BCVA: best-corrected visual acuity

Next, we evaluated the correlation of VL, BD, and VL/BD with BCVA and CMT. We did not detect any significant correlation of VL and BD with BCVA, CMT, and FAZ (data not shown). However, VL/BDs were correlated with the same postoperative BCVA at 1, 3, and 6 months (coefficient = 0.318, 0.319 and 0.307, *p* = 0.035, 0.035, and 0.042, respectively) in the superior-inferior quadrant (Table 3, Fig. 3a).

**Table 3:** Factors associated with VL/BD in the univariate analysis

	Temporal		Nasal		Superior + Inferior	
	Coefficient	*p* value	Coefficient	*p* value	Coefficient	*p* value
BCVA						
1 M	− 0.046	0.838	− 0.143	0.525	0.318	0.035
3 M	− 0.012	0.959	0.115	0.61	0.319	0.035
6 M	−0.05	0.827	0.033	0.885	0.307	0.042
CMT						
1 M	0.14	0.534	0.257	0.249	0.075	0.627
3 M	0.444	0.044	0.058	0.803	0.144	0.364
6 M	0.382	0.088	−0.186	0.421	0.074	0.641
FAZ						
1 M	−0.31	0.16	−0.26	0.242	−0.001	0.998
3 M	−0.295	0.183	0.042	0.854	0.029	0.853
6 M	−0.263	0.236	0.174	0.438	0.095	0.541

*VL* Actual vessel length, *BD* Direct vessel branching point distance, *BCVA* Best corrected visual acuity, *CMT* Central macular thickness, *FAZ* Foveal avascular zone, *1 M* 1 month, *3 M* 3 months, *6 M* 6 months

Finally, we analyzed the correlation of VL change with changes in BCVA, CMT, and FAZ. We detected a significant association between the changes in VL and BCVA at 3 months (coefficient = 0.497, *p* = 0.0189) and 6 months (coefficient = 0.423, *p* = 0.0455) postoperatively in the nasal quadrant (Table 4, Fig. 3b), even though there was a significant association between the changes in VL and baseline BCVA (Table 4).

**Table 4:** Factors associated with the change in the actual vascular length in univariate analysis

	Temporal		Nasal		Superior + Inferior	
	Coefficient	*p* value	Coefficient	*p* value	Coefficient	*p* value
Preoperative BCVA						
1 M	−0.001	0.996	−0.436	0.042	0.133	0.389
3 M	0.046	0.84	−0.489	0.021	0.014	0.926
6 M	−0.094	0.677	−0.471	0.027	−0.038	0.805
ΔBCVA						
1 M	−0.173	0.442	0.343	0.118	−0.2	0.193
3 M	−0.188	0.402	0.497	0.0189	−0.182	0.238
6 M	−0.04	0.861	0.423	0.0455	−0.017	0.911
ΔCMT						
1 M	−0.544	0.009	0.116	0.608	−0.182	0.238
3 M	−0.422	0.057	0.373	0.096	−0.272	0.082
6 M	−0.296	0.193	0.384	0.086	−0.34	0.027
ΔFAZ						
1 M	0.174	0.438	0.058	0.796	0.193	0.211
3 M	0.21	0.348	0.015	0.949	0.199	0.194
6 M	0.088	0.696	0.12	0.595	0.193	0.21

*BCVA* Best corrected visual acuity, *CMT* Central macular thickness, *FAZ* Foveal avascular zone, *1 M* 1 month, *3 M* 3 months, *6 M* 6 months

## Discussion

In the current study, we measured the distortion of retinal blood vessels around the fovea to evaluate the transition of tractional force on ERM after surgery. Shrinkage of retinal vessels was calculated as the ratio of VL and BD (VL/BD) between any two bifurcations. VL/BDs were significantly decreased in the superior, inferior, and temporal quadrants and tended to be in the nasal quadrant during the postoperative period. A decrease in VL/BD indicates that vessels have become more linearized due to the release of tractional force generated by the ERM, such that the tangential tractional force was centrifuged. We also found a statistically significant correlation between better postoperative visual acuity and VL/BD in the superior-inferior quadrant. Furthermore, changes in visual acuity after surgery were correlated with changes in the actual VL in the nasal quadrant.

To date, studies on individual retinal vessels in eyes with ERM before and after surgery have not been performed. Kofold et al. measured the movement of vasculature using an infrared fundus picture but not the distortion of vessels [12]. Retinal vessel movements correlated with decreased BCVA and increased CMT were more common in patients with worsening symptoms. Momota et al. quantified the distance between the retinal vessel bifurcations and the fovea using OCTA in eyes with ERM following surgery [16]. They demonstrated that significant retinal displacement occurred centrifugally and asymmetrically in the four quadrants postoperatively. However, as demonstrated in this study, changes in FAZ due to the release of ERM traction should have affected these results. Kim et al. measured the length of the radial vessel segment (VLA) and the length from the foveola to the vessel branching point (FBL) using infrared fundus photographs [17]. The FBL of the superior and inferior areas significantly increased postoperatively. Furthermore, a positive correlation was observed between the differences in FBL and macular thickness in the superior area. However, postoperative changes in VLA and FBL did not show a significant correlation with postoperative BCVA and BCVA differences. The measurement of VL in this study was based on the fovea; hence, changes in FAZ should have influenced the results as well. In our study, the VL/BD significantly decreased after surgery, suggesting that after ERM removal, the actual VL becomes shorter and/or the BD between two bifurcations becomes longer. Moreover, these findings reveal that the vessels are not linearized but tortuous in eyes with ERM.

Centripetal tractional force is believed to be the main pathogenesis of ERM [16, 18]. In the current study, a decrease in VL/BD was found in the temporal and superior-inferior quadrants postoperatively. VL/BD represents the division of the distance of a specific vessel by the one-line distance between the edges of the vessel. Thus, a decrease in VL/BD indicates that the vessels become more linear in shape and that traction by ERM made the vessels more tortuous. Moreover, the result that VL in the temporal quadrant became significantly longer postoperatively indicates that the shrunken vessels extended after the release of ERM traction.

Nevertheless, in the nasal quadrant, VL/BD did not show any significant change and VL significantly decreased after surgery. One possible reason could be that the vessels in the nasal cavity were not affected by the presence of optic disc. The distal end to the macula and proximal end to the optic disc might not be affected by ERM traction due to the presence of the optic disc. This resulted in more linear vessels in the nasal quadrant, although vessels in the other three quadrants were more tortuous.

Several groups have reported retinal vascular changes associated with visual outcomes after ERM surgery. Yang et al. showed that the area enclosed by the superior and inferior major vessels from the optic disc to the fovea (area under major vessel [AUV]) decreased in eyes with ERM and correlated with preoperative visual acuity and CMT [19]. Although improvement of visual acuity did not correlate with the difference in AUV, postoperative visual acuity was associated with AUV [19]. Furthermore, Rodrigues et al. reported that the vertical distance between the arcade vessels, termed the interarcade distance, increased after ERM surgery [20]. In the present study, better postoperative visual acuity was correlated with VL/BD in the superior-inferior quadrants. These findings indicate that vessel tortuosity superior or inferior to the macula represents postoperative visual acuity during ERM surgery.

The limitations of this study include the inclusion of several points. First, many of the eyes included in this study underwent simultaneous cataract surgery. This limitation has been included in previous studies as well. Second, because the sample size was relatively small, randomly selecting a vessel in each quadrant might have led to a bias in the analysis. Third, even though the study was performed in a blinded fashion, the vessels were selected manually. Furthermore, technologies that can calculate object tortuosity may have been required in the current study.

## Conclusions

Using OCTA, we were able to detect changes in vascular distortion after ERM surgery. In the current study, the vessels became more linear due to ERM removal, except in the nasal quadrant. The vessels became longer in the temporal quadrant, and VL in the nasal quadrant decreased after surgery. Furthermore, we inferred that superior and nasal vascular changes are associated with visual outcomes in eyes that underwent ERM surgery. Severe retinal traction, represented by vascular strain and quantified by OCTA utilization, is potentially associated with poor visual outcomes in ERM surgery.

## Data Availability

All data generated or analyzed during this study are included in this article. Further enquiries can be directed to the corresponding author.

## Abbreviations

AUV: Area under major vessel
BCVA: Best-corrected visual acuity
BD: Direct vessel branching point distance
CMT: Central macular thickness
ERM: Epiretinal membrane
FAZ: Foveal avascular zone
OCTA: Optical coherence tomography angiography
VL: The actual vessel length
